# The lipid accumulation product is a powerful tool to diagnose metabolic dysfunction-associated fatty liver disease in the United States adults

**DOI:** 10.3389/fendo.2022.977625

**Published:** 2022-11-04

**Authors:** Hejun Li, Ying Zhang, Hengcong Luo, Rong Lin

**Affiliations:** Department of Endocrinology, The Third Affiliated Hospital of Guangzhou Medical University, Guangzhou, China

**Keywords:** NHANES, MAFLD, United States, lipid accumulation product (LAP), cross-sectional study

## Abstract

**Background & objectives:**

Body mass index (BMI) and waist circumference (WC) are widely used to assess obesity, but they are limited in their ability to distinguish complicated body metabolic situations (fat mass, lean body mass, visceral and subcutaneous fat deposits in the abdomen). The purpose of this study was to evaluate the diagnostic efficacy of different anthropometric indices in metabolic dysfunction-associated fatty liver disease (MAFLD) and to identify the best cut-off point for the diagnosis of MAFLD in United States adults.

**Methods:**

A cross-sectional study among 4,195 participants over 18 years old in the National Health and Nutrition Examination Survey (NHANES) 2017–2018 was performed. All patients underwent vibration controlled transient elastography (VCTE). Assess the anthropometric measurements, including BMI, WC, waist-to-height ratio (WHtR), waist-to-hip ratio (WHR), cardiometabolic index (CMI), triglyceride-glucose (TyG) index, hepatic steatosis index (HSI), lipid accumulation product (LAP), body roundness index (BRI), visceral fat index (VAI), abdominal volume index (AVI), cone index (CI), and body fat index (BAI). Logistic regression analyses were conducted to estimate the impact of these indices, on the odds ratio (OR) values of MAFLD. Receiver operator characteristic (ROC) analyses were performed to assess the diagnosing capacity of these anthropometric indices for MAFLD and identify the optimal cut-offs points.

**Results:**

A total of 4,195 (2,069 men and 2,126 women) participants were performed, with 45.4 ± 0.64 (mean ± SD) years old. All anthropometric metrics were positively associated with MAFLD, irrespective of whether it was treated as continuous or categorical variable (P<0.05). Multivariate logistic regression showed a positive correlation between AVI, HSI, WHtR, BRI, and MAFLD, with significant interaction with gender. ROC curves results showed that LAP had the highest AUC [0.813 (95% CI, 0.800–0.826)], especially in participants aged between 18 and 50 years old. Furthermore, LAP showed the highest ROC in both the training set [0.812 (95% CI, 0.800–0.835)] and the validation set [0.809 (95% CI, 0.791–0.827)].

**Conclusions:**

In the present study, we showed that those anthropometric indices were significantly associated with MAFLD in United States adults. Besides, the association of HSI, BRI, AVI, and WHtR with MAFLD was more obvious in men than in women. LAP may be a sensitive marker for diagnosing MAFLD in U.S. adults.

## Introduction

Non-alcoholic fatty liver disease (NAFLD) has become the most prevalent chronic liver disease in western countries, owing to its increasing incidence and an aging population (1). The global prevalence of NAFLD has increased from affecting nearly a quarter of people in or before 2005 to more than a third by 2016 or later (2). Meanwhile, due to its high prevalence, NAFLD is now a leading cause of end-stage liver disease and liver cancer, which imposes a major economic burden on society and families (3, 4). With the advanced understanding of NAFLD, it has been found that NAFLD is closely associated with metabolic dysfunction and is also an independent risk factor for a range of cardiovascular and metabolic diseases, such as hypertension, insulin resistance, type 2 diabetes mellitus (T2DM), dyslipidemia, and hypertriglyceridemia (5, 6).

Therefore, an international panel of experts has recently proposed a consensus on updating the nomenclature for metabolic dysfunction-associated fatty liver disease (MAFLD) (7, 8). A growing number of studies confirm that patients with MAFLD have more metabolic disorder traits than those with NAFLD (9, 10). In addition, several studies have further demonstrated that patients with MAFLD have more severe extra-hepatic organ diseases, such as chronic kidney disease, chronic cardiovascular disease, and even increased all-cause mortality (11–13). This means that there is a considerable clinical difference between the MAFLD population and the NAFLD population.

Obesity is closely associated with the incidence of MAFLD, and body mass index (BMI) is often used to assess overall obesity (14). Nonetheless, nearly a sixth of the population with MAFLD was classified as lean, and around 40% of the NAFLD population was without obesity (15, 16). These people are known as metabolically unhealthy patients without obesity. Evidence in the literature supports the standpoint that these lean patients with NAFLD have even worse clinical outcomes than NAFLD individuals with obesity (17, 18). This may be attributed to the fact that these individuals without obesity may actually have body components that favor visceral fat obesity and insulin resistance, thus contributing to the development of NAFLD (19).

In summary, it is difficult to identify patients with MAFLD early and accurately based on BMI alone, so it is necessary to find better indicators of central obesity and insulin resistance (IR) to assist in the diagnosis of MAFLD. Easy-to-calculate and accessible anthropometric indicators such as lipid accumulation product (LAP), body roundness index (BRI), visceral fat index (VAI), cone index (CI), and body fat index (BAI) have been used as proxies for IR and central obesity and can quantify visceral fat status (20–22). In addition, the CMI, triglyceride-glucose (TyG) index, and hepatic steatosis index (HSI) have been confirmed to reflect adiposity and IR (23–25).

Therefore, the purpose of this study was to evaluate the diagnostic efficacy of these indicators (BMI, waist circumference (WC), waist-to-height ratio (WHtR), waist-to-hip ratio (WHR), CMI, TyG index, HSI, LAP, BRI, VAI, AVI, CI, and BAI) as well as find the optimal cut-off points for the diagnosis of MAFLD in American adults.

## Methods

### Study participants

NHANES is a cross-sectional, nationally representative survey that collects health examination data using a stratified, multistage probability design to select a representative sample of the non-institutionalized population of the United States. The data included health interviews, examination components, and laboratory tests administered by highly trained medical personnel. In the current study, we included participants who participated in the 2017–2018 NHANES study cycles. Because all participants had signed written informed consent in the original survey and their personal information was fully de-identified, our study was granted an exemption from the Institutional Review Board.

**Figure 1:**
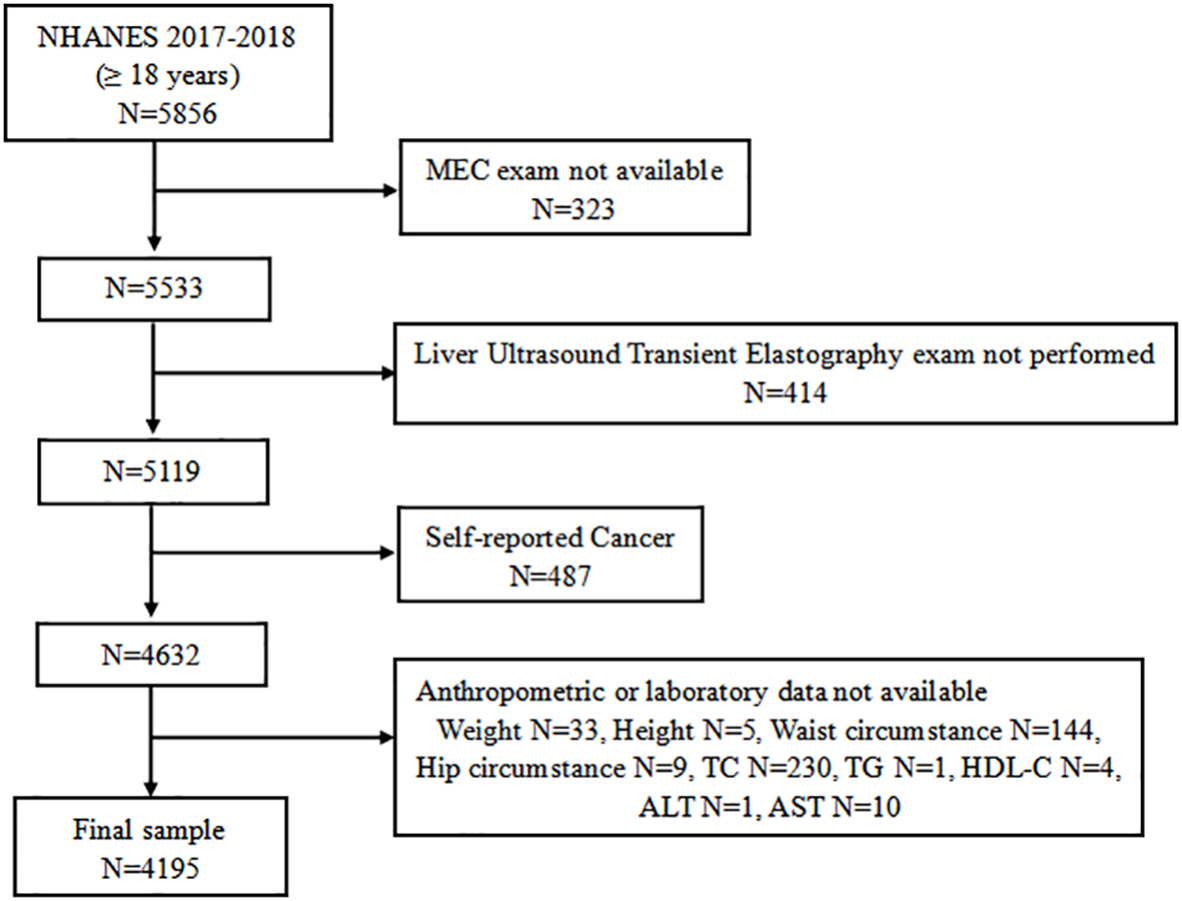
Flow chart of the research study design.

A total of 9,254 individuals participated in the 2017–2018 NHANES cycles. The study population for the current analysis consisted of adolescents aged 18 years and older who attended a Mobile Exam Center (MEC) (n = 5,533). We initially excluded 414 individuals who did not attend vibration-controlled transient elastography (VCTE). An additional 487 individuals were excluded from the analyses as self-reported cancer patients. Of the remaining 4,632 participants, 437 who lacked complete anthropometric or laboratory data were excluded. Finally, a total of 4,195 subjects were eligible for further analysis (**Figure 1**).

### Clinical and laboratory data

The demographic information such as age (years), gender (male or female), race/ethnicity (Mexican American, Other Hispanic, Non-Hispanic White, Non-Hispanic Black, and Other Race), educational level (Below high school, high school, and above), and marital status (Married/Living with partner, Widowed/Divorced/Separated/Never married) were ascertained by the questionnaire. Standing height (cm), weight (kg), waist circumference (cm), and hip circumference (cm) were measured by anthropometric measurement in the MEC.

After a night fast of at least 8 h, blood samples were collected. Serum was tested for fasting blood glucose (mg/dl), hemoglobin A1C (%), total cholesterol (mg/dl), high-density lipoprotein (HDL) cholesterol (mg/dl), low-density lipoprotein (LDL) cholesterol (mg/dl), triglyceride (mg/dl), high-sensitivity C-reactive protein (mg/L), alanine aminotransferase (ALT) (IU/L), aspartate aminotransferase (AST) (IU/L), alkaline phosphatase (ALP) (IU/L), gamma glutamyl transferase (GGT) (IU/L), total bilirubin (mg/dl), creatinine (mg/dl), albumin (g/dl), and uric acid (mg/dl).

Blood pressure is measured by a blood pressure inspector who has passed an accredited training program. Participants were measured three times after sitting for 5 min to determine the maximum dilation level (MIL). We took the average of the three measurements as the representative blood pressure of the participants. Hypertension was defined by the presence of one of two criteria: systolic blood pressure ≥130 mmHg and/or diastolic blood pressure ≥85 mmHg or currently taking antihypertensive medications. Diabetes mellitus was defined by the presence of one of the following conditions: a) self-reported diabetes; b) fasting blood glucose ≥126 mg/dl; c) HBA1c level ≥6.5%; and d) use of the anti-diabetic drug, including insulin (26).

### Vibration controlled transient elastography

In the 2017–2018 cycle of NHANES, eligible participants underwent liver VCTE measurement using the FibroScan 502 V2 Touch (Echosens). Liver steatosis was defined by the controlled attenuation parameter (CAP), with a cut-off of 274 dB/m. In a recent report (27), this cut-off showed 90% sensitivity in identifying any degree of hepatic steatosis.

### MAFLD definition

According to the 2020 International Expert Consensus Statement (8), a positive diagnosis of MAFLD is based on VCTE evidence of fat accumulation in the liver (hepatic steatosis) in addition to one of the following three criteria: overweight/obesity; presence of T2DM; or evidence of metabolic dysregulation. Metabolic dysregulation exhibits the following characteristics: 1) waist circumference ≥102/88 cm in Caucasian men and women; 2) blood pressure ≥130/85 mmHg or specific drug treatment; 3) plasma triglycerides ≥150 mg/dl or specific drug treatment; 4) plasma HDL-cholesterol<40 mg/dl for men and<50 mg/dl for women or specific drug treatment; 5) prediabetes (fasting glucose levels 100 to 125 mg/dl, or HbA1c 5.7% to 6.4%; 6) HOMA-IR score ≥2.5; and 7) plasma high-sensitivity C-reactive protein level >2 mg/L.

### Anthropometric index calculation


$$\text{BMI}=\frac{Weight_{\left(kg\right)}}{Heigh{t_{\left(m\right)}}^{2}}$$

$$\text{WHtR}=\frac{WC_{\left(cm\right)}}{Height_{\left(cm\right)}}$$

$$\text{WHR}=\frac{WC_{\left(cm\right)}}{HC_{\left(cm\right)}}$$

$\text{LAP}=({\mathrm{WC}}_{\left(\mathrm{cm}\right)}-65)\times {\mathrm{TG}}_{\left(\mathrm{mmol}/L\right)}$ in males;

$\text{LAP}=({\mathrm{WC}}_{\left(\mathrm{cm}\right)}-58)\times {\mathrm{TG}}_{\left(\mathrm{mmol}/L\right)}$ in females (28).

$\text{CMI}=WHtR\times \left(\frac{TG_{\left(mmol/L\right)}}{HDL_{\left(mmol/L\right)}}\right)$ (23)

$\text{BRI}=364.2-365.5\times \sqrt{1-{\left(\frac{\frac{WC_{\left(m\right)}}{2\pi }}{0.5\times Height_{\left(m\right)}}\right)}^{2}}$ (29).

$\text{TyG index}=Ln\;\left[\frac{TG_{\left(mg/dL\right)}\times FPG_{\left(mg/dL\right)}}{2}\right]$ (25)

$\text{CI}=\frac{WC_{\left(m\right)}}{0.109\times \sqrt{\frac{Weight_{\left(kg\right)}}{Height_{\left(cm\right)}}}}$ (30).

$\text{BAI}=\frac{HC_{\left(cm\right)}}{Heigh{t_{\left(m\right)}}^{\frac{3}{2}}}-18$ (31).

$\text{AVI}=\frac{2\times {\left(WC_{\left(cm\right)}\right)}^{2}+0.7\times {\left(WC_{\left(cm\right)}-HC_{\left(cm\right)}\right)}^{2}}{1000}$ (32).

$\text{HSI}=8\times \left(\frac{ALT}{AST}\right)+BMI\left(+2,if\;female;+2,if\;diabetes\;millitus\right)$ (24).

$\text{VAI}=\left(\frac{WC_{\left(cm\right)}}{39.68+\left(1.88\times BMI\right)}\right)\times \left(\frac{TG_{\left(mmol/L\right)}}{1.03}\right)\times \left(\frac{1.31}{HDL_{\left(mmol/L\right)}}\right)\;$ *in males*,

$\text{VAI}=\left(\frac{WC_{\left(cm\right)}}{36.58+\left(1.89\times BMI\right)}\right)\times \left(\frac{TG_{\left(mmol/L\right)}}{0.81}\right)\times \left(\frac{1.52}{HDL_{\left(mmol/L\right)}}\right)$ *in females* (33).

### Statistical analysis

In accounting for the complex survey design of NHANES, we used appropriate weighting for each analysis, as suggested by the NCHS. Continuous variables were presented as weighted average means with standard deviation (SD), and categorical variables were presented as weighted numbers with percentages (%). The Chi-square test was performed for categorical variables and the Rao–Scott χ^2^ test for continuous variables to describe the clinical characteristics of patients with or without MAFLD. Weighted multiple linear or logistic regression analyses were conducted to analyze the association between those obesity indices and MAFLD after adjusting for known or selected confounders in different models. In model I, no covariates were adjusted. Model II was adjusted for gender, age, race, education level, and marital status. Model 3 was adjusted by model II plus uric acid. To allow direct comparison of odds ratio (OR) values, those obesity and lipid-related indices were converted into Z-scores. Each anthropometric index was assessed using quartiles, comparing each of the upper 3 quartiles with the lowest quartile (reference). Subgroup analysis stratified by gender, age, BMI, and abdominal obesity was also performed by stratified multivariate regression analysis. To compare the diagnostic power of those obesity and lipid-related indices for MAFLD, the area under receiver operating characteristic (ROC) curves was employed to evaluate the abilities of the anthropometric indices to diagnose MAFLD. The cutoff value was selected based on the highest Youden’s index in order to maximize both sensitivity and specificity (Youden’s index = sensitivity + specificity − 1). All analyses were conducted using R version 3.6.1 (R Foundation for Statistical Computing, Vienna, Austria), with statistical significance being identified at the level of P<0.05.

## Results

### Baseline characteristics of the participants with and without MAFLD

The cross-sectional analysis included 4195 participants (median age was 45.4 years; 49.6% were male). The clinical characteristics of participants with and without MAFLD are presented in **Table 1**. Participants with MAFLD were significantly older, more frequently overweight, and had abdominal obesity, with no significant differences in sex distribution. Compared with participants without MAFLD, those with MAFLD were characterized by worse metabolic disease, as demonstrated by diabetes, prediabetes, insulin resistance, and hypertension. Additionally, these participants with MAFLD were noted to have a higher waist circumference, hip circumference, hs-CRP, ALT, GGT, Bun, albumin, uric acid, anthropometric indices, and lower HDL. Male participants in the group with MAFLD had a higher TC and total bilirubin. Females with MAFLD had more elevated AST and ALP.

**Table 1:** Clinical characteristics of the study participants classified by the presence of different gender and metabolic dysfunction-associated fatty liver disease (MAFLD).

	Total	Male			Female		
		MAFLD	Non-MAFLD	P-value	MAFLD	Non-MAFLD	P-value
N	4,195	1,072	997		1,330	796	
Age (years)	45.4 ± 0.64	47.87 ± 0.82	41.67 ± 1.02	<0.001***	50.58 ± 0.73	43.86 ± 0.75	<0.001***
Race/ethnicity (%)				<0.001***			<0.01**
Mexican American	9.78 ± 1.75	14.92 ± 3.03	7.30 ± 1.42		12.13 ± 2.26	6.87 ± 1.31	
Other Hispanic	7.15 ± 0.83	5.67 ± 0.82	7.59 ± 1.27		7.66 ± 1.26	7.55 ± 1.07	
Non-Hispanic White	60.96 ± 2.55	60.5 ± 3.49	61.31 ± 2.96		58.38 ± 3.06	62.43 ± 2.93	
Non-Hispanic Black	11.03 ± 1.64	7.71 ± 1.53	12.56 ± 1.84		11.24 ± 1.85	12.01 ± 1.71	
Mixed non‐Hispanic	11.09 ± 1.35	11.21 ± 1.64	11.25 ± 1.62		10.59 ± 2.04	11.15 ± 1.68	
Education level (%)				0.9171			<0.01**
High school and below	38.85 ± 1.88	41.21 ± 2.71	41.58 ± 3.64		41.49 ± 1.75	33.41 ± 2.56	
Higher than high school	61.15 ± 1.88	58.79 ± 2.71	58.42 ± 3.64		58.51 ± 1.75	66.59 ± 2.56	
Marital status (%)				<0.001***			0.0852
Married or living with partner	62.48 ± 1.48	72.96 ± 2.59	58.45 ± 1.19		63.95 ± 2.65	57.23 ± 2.81	
Widowed/Divorced/Separated/Never married	37.52 ± 1.48	27.04 ± 2.59	41.55 ± 1.19		36.05 ± 2.65	42.77 ± 2.81	
Lipid lowering medication use (%)	25.53 ± 1.44	38.19 ± 2.54	14.93 ± 2.10	<0.001***	39.15 ± 3.18	17.73 ± 1.27	<0.001***
Antihypertensive medication use (%)	22.16 ± 1.17	34.02 ± 2.13	15.26 ± 2.43	<0.001***	28.95 ± 2.73	15.66 ± 1.31	<0.001***
Height (cm)	168.24 ± 0.27	175.6 ± 0.32	175.03 ± 0.35	0.1546	161.05 ± 0.42	161.41 ± 0.20	0.3903
Weight (kg)	84.02 ± 0.84	102.18 ± 1.36	80.81 ± 0.77	<0.001***	91.05 ± 1.32	69.91 ± 0.95	<0.001***
Body mass index (kg/m^2^)	29.58 ± 0.3	33.02 ± 0.43	26.33 ± 0.27	<0.001***	35.03 ± 0.54	26.8 ± 0.35	<0.001***
Waist circumference (cm)	100.09 ± 0.79	112.65 ± 1.13	93.45 ± 0.77	<0.001***	110.53 ± 1.1	90.87 ± 0.93	<0.001***
Hip circumference (cm)	107.34 ± 0.50	111.43 ± 0.8	100.11 ± 0.43	<0.001***	118.92 ± 1.01	103.98 ± 0.60	<0.001***
Hypertension (%)	39.94 ± 1.65	57.69 ± 3.10	30.03 ± 1.80	<0.001***	51.39 ± 2.98	29.17 ± 1.90	<0.001***
Diabetes (%)	13.46 ± 0.58	23.74 ± 1.07	6.27 ± 1.22	<0.001***	24.4 ± 2.17	6.03 ± 1.00	<0.001***
Prediabetes (%)	34.51 ± 1.10	47.03 ± 3.00	26.8 ± 2.32	<0.001***	48.5 ± 2.27	24.26 ± 1.53	<0.001***
Overweight (%)	72.14 ± 1.42	95.47 ± 1.20	59.53 ± 2.86	<0.001***	95.53 ± 1.03	53.06 ± 2.32	<0.001***
Abdominal obesity (%)	57.76 ± 1.81	76.67 ± 2.38	24.31 ± 2.95	<0.001***	94.58 ± 0.97	51.51 ± 2.47	<0.001***
Hypertriglyceridemia (%)	32.47 ± 1.58	55.3 ± 2.90	24.99 ± 2.40	<0.001***	44.94 ± 1.85	15.55 ± 2.01	<0.001***
Low HDL (%)	49.43 ± 1.26	87.2 ± 1.65	58.6 ± 1.84	<0.001***	41.37 ± 3.25	19.59 ± 1.48	<0.001***
Insulin resistance (%)	20.93 ± 0.86	33.73 ± 2.63	12.15 ± 1.76	<0.001***	33.47 ± 3.86	12.15 ± 1.39	<0.001***
High sensitivity CRP (%)	46.41 ± 1.69	54.22 ± 2.11	29.32 ± 2.11	<0.001***	73.44 ± 2.22	39.95 ± 2.96	<0.001***
Drinking status (%)				0.96			0.275
Never drinkers	7.57 ± 0.61	6.23 ± 1.43	5.92 ± 0.83		10.31 ± 2.06	8.39 ± 1.15	
Current drinkers	77.02 ± 0.97	78.45 ± 2.21	78.28 ± 1.96		72.27 ± 2.16	77.57 ± 1.57	
Former drinkers	15.41 ± 0.94	15.32 ± 1.67	15.8 ± 1.67		17.42 ± 1.67	14.04 ± 1.37	
Smoking status (%)				<0.01**			0.3686
Never smokers	59.17 ± 1.59	50.51 ± 2.49	51.2 ± 2.46		64.99 ± 2.73	68.64 ± 1.83	
Current smokers	17.11 ± 1.16	14.7 ± 1.35	22.01 ± 1.92		17.45 ± 1.91	14.6 ± 1.61	
Former smokers	23.72 ± 1.08	34.79 ± 2.83	26.79 ± 2.23		17.56 ± 2.25	16.75 ± 1.12	
Hepatitis (%)	2.73 ± 0.44	3.1 ± 0.82	3.82 ± 1.30	0.6614	1.76 ± 0.58	2.1 ± 0.85	0.7415
Lipid profile (mg/dl)							
Total cholesterol	188.4 ± 1.65	191.11 ± 2.44	181.84 ± 2.12	<0.01**	194.46 ± 3.00	188.51 ± 1.86	0.0548
Triglyceride	140.6 ± 3.27	197.25 ± 7.78	123.14 ± 3.43	<0.001***	159.39 ± 4.19	104.42 ± 2.63	<0.001***
High-density cholesterol	53.34 ± 0.51	43.99 ± 0.70	51.91 ± 0.38	<0.001***	53.06 ± 0.79	61.3 ± 0.73	<0.001***
Low-density cholesterol	110.5 ± 1.58	113.76 ± 2.93	107.82 ± 1.87	0.0585	114.09 ± 4.6	108.76 ± 2.16	0.3542
Systolic blood pressure (mmHg)	121.12 ± 0.40	126.37 ± 0.65	122.08 ± 0.45	<0.001***	120.88 ± 0.99	116.6 ± 0.77	<0.01**
Diastolic blood pressure (mmHg)	74.17 ± 0.38	78.44 ± 0.74	73.37 ± 0.56	<0.001***	75.16 ± 0.67	71.14 ± 0.44	<0.001***
ALT (IU/L)	23.53 ± 0.46	33.13 ± 1.19	23.85 ± 1.16	<0.001***	22.27 ± 0.68	17.16 ± 0.41	<0.001***
AST (IU/L)	22.42 ± 0.30	25.26 ± 0.68	23.93 ± 1.05	0.3302	21.4 ± 0.56	19.75 ± 0.31	<0.05*
GGT (IU/L)	29.88 ± 0.67	43.27 ± 1.58	29.39 ± 1.86	<0.001***	29.73 ± 1.22	20.89 ± 1.21	<0.001***
ALP (IU/L)	76.47 ± 0.64	77.83 ± 1.34	75.48 ± 1.29	0.2854	82.66 ± 1.37	72.89 ± 1.32	<0.001***
Total bilirubin (mg/dl)	0.47 ± 0.01	0.51 ± 0.02	0.59 ± 0.02	<0.05*	0.37 ± 0.01	0.4 ± 0.01	0.0604
Albumin (g/dl)	4.11 ± 0.02	4.16 ± 0.02	4.24 ± 0.02	<0.05*	3.95 ± 0.02	4.05 ± 0.02	<0.001***
Bun (mg/dl)	14.51 ± 0.17	15.72 ± 0.26	15.13 ± 0.18	<0.05*	14.14 ± 0.33	13.35 ± 0.17	<0.05*
Serum creatinine (mg/dl)	0.87 ± 0.01	0.99 ± 0.01	0.99 ± 0.01	0.9387	0.75 ± 0.01	0.76 ± 0.01	0.7619
Uric acid (mg/dl)	5.37 ± 0.03	6.29 ± 0.04	5.83 ± 0.07	<0.001***	5.19 ± 0.06	4.44 ± 0.05	<0.001***
Obesity-related indices							
WHtR	0.6 ± 0	0.64 ± 0.01	0.53 ± 0	<0.001***	0.69 ± 0.01	0.56 ± 0.01	<0.001***
WHR	0.93 ± 0	1.01 ± 0	0.93 ± 0	<0.001***	0.93 ± 0	0.87 ± 0	<0.001***
LAP	66.31 ± 2.78	106.21 ± 5.57	42.5 ± 2.05	<0.001***	96.12 ± 3.9	41.12 ± 1.93	<0.001***
CMI	0.85 ± 0.03	1.45 ± 0.08	0.66 ± 0.03	<0.001***	1.05 ± 0.04	0.48 ± 0.02	<0.001***
BRI	5.55 ± 0.11	6.53 ± 0.16	4.13 ± 0.10	<0.001***	7.73 ± 0.2	4.82 ± 0.13	<0.001***
TyG index	8.65 ± 0.02	9.07 ± 0.04	8.5 ± 0.02	<0.001***	8.87 ± 0.03	8.36 ± 0.02	<0.001***
CI	0.13 ± 0	0.14 ± 0	0.13 ± 0	<0.001***	0.14 ± 0	0.13 ± 0	<0.001***
BAI	31.49 ± 0.28	29.94 ± 0.36	25.3 ± 0.25	<0.001***	40.29 ± 0.57	32.8 ± 0.30	<0.001***
AVI	20.74 ± 0.33	25.83 ± 0.52	17.84 ± 0.29	<0.001***	25.03 ± 0.51	17.15 ± 0.36	<0.001***
HSI	39.05 ± 0.38	43.8 ± 0.56	34.42 ± 0.37	<0.001***	45.75 ± 0.66	35.78 ± 0.40	<0.001***
VAI	2.23 ± 0.07	3.15 ± 0.17	1.6 ± 0.06	<0.001***	3.06 ± 0.11	1.62 ± 0.06	<0.001***

Continuous data are shown as the mean ± SD and categorical data as n (%).

ALT, alanine aminotransferase; AST, aspartate aminotransferase; GGT, gamma glutamyl transferase; ALP, alkaline phosphatase; BUN, blood urea nitrogen; WHtR, waist-to-height ratio; WHR, waist-to-hip ratio; LAP, lipid accumulation product; CMI, cardiometabolic index; BRI, body roundness index; TyG index, triglyceride-glucose (TyG) index; CI, conicity index; BAI, body adiposity index; AVI, abdominal volume index; HSI, hepatic steatosis index; VAI, visceral adiposity index.

*P-value<0.05; **P-value<0.01; ***P-value<0.001.

### Associations between various baseline anthropometric indices and MAFLD

Univariate logistic regression analysis showed that BMI, WHtR, WHR, LAP, HSI, AVI, BAI, BRI, CI, CMI, TyG index, and VAI were positively associated with MAFLD (P<0.001) (**Table 2**). After adjusting for all covariates (model 3), the multiple logistic regression analyses presented that all anthropometric indices, including BMI (OR = 3.44, 95% CI: 3.03 to 3.90), LAP (OR = 3.79, 95% CI: 3.25 to 4.40), AVI (OR = 3.54, 95% CI: 3.12 to 4.03), HSI (OR = 4.12, 95% CI: 3.61 to 4.71), BRI (OR = 2.22, 95% CI: 2.00 to 2.47), and WHtR (OR = 3.64, 95% CI: 3.20 to 4.14), were positively associated with MAFLD (**Table 3**), since all anthropometric indicators were translated to Z-scores before multiple logistic regression analysis. LAP showed a high OR value in model 3, which may suggest that LAP is a better index for diagnosing MAFLD. Multiple logistic regression analysis was then performed to evaluate the odds ratio values of different anthropometric indicators in MAFLD patients. In model 3, participants with an elevated LAP (LAP ≥82.579) (Q4) were positively associated with a MAFLD compared with their counterparts whose LAP was<26.157 (Q1). Similar results were observed in other anthropometric indices.

**Table 2:** Univariate logistic regression models evaluating the association of demographic, biochemical and clinical characteristics, and anthropometric indexes with MAFLD (Per SD increment for continuous variables).

	HR, 95% CI(Per SD increment for continuous variables)	P-value
Age (years)	1.57 (1.43, 1.73)	<0.001***
Male	0.64 (0.56, 0.73)	<0.001***
TG (mg/dl)	2.82 (2.43, 3.27)	<0.001***
TC (mg/dl)	1.21 (1.09, 1.34)	<0.01**
HDL (mg/dl)	0.48 (0.42, 0.55)	<0.001***
LDL (mg/dl)	1.18 (0.96, 1.43)	0. 1328
Glu (mg/dl)	2.65 (2.21, 3.17)	<0.001***
UA (mg/dl)	1.77 (1.56, 2.01)	<0.001***
Bun (mg/dl)	1.23 (1.12, 1.35)	<0.001***
Scr (mg/dl)	1.12 (1.02, 1.23)	<0.05*
SBP (mmHg)	1.39 (1.25, 1.54)	<0.001***
DBP (mmHg)	1.64 (1.46, 1.84)	<0.001***
ALT (IU/L)	1.72 (1.37, 2.16)	<0.001***
AST (IU/L)	1.14 (1.01, 1.29)	0.0521
GGT (IU/L)	1.63 (1.27, 2.10)	<0.01**
ALP (IU/L)	1.68 (1.29, 2.19)	<0.01**
Tbil (mg/dl)	0.89 (0.78, 1.01)	0.0961
ALB (g/dl)	0.81 (0.73, 0.91)	<0.01**
Weight (kg)	3.67 (3.15, 4.89)	<0.001***
BMI (kg/m²)	4.06 (3.37, 4.89)	<0.001***
WC (cm)	5.05 (4.25, 5.99)	<0.001***
HC (cm)	3.11 (2.73, 3.55)	<0.001***
WHtR	4.31 (3.62, 5.13)	<0.001***
WHR	3.20 (2.73, 3.76)	<0.001***
LAP	5.86 (4.98, 6.90)	<0.001***
his	4.53 (3.81, 5.39)	<0.001***
AVI	4.72 (3.91, 5.71)	<0.001***
BAI	2.03 (1.86, 2.20)	<0.001***
BRI	4.05 (3.35, 4.89)	<0.001***
CI	3.52 (2.90, 4.27)	<0.001***
CMI	4.65 (3.90, 5.54)	<0.001***
TyG index	3.05 (2.68, 3.46)	<0.001***
VAI	3.31 (2.89, 3.79)	<0.001***

TG, triglycerides; TC, total cholesterol; HDL, high-density lipoprotein; LDL, low-density lipoprotein; Glu, glucose; UA, urid acid; Bun, blood urea nitrogen; Scr, serum creatinine; SBP, systolic blood pressure; DBP, diastolic blood pressure; ALT, alanine aminotransferase; AST, aspartate aminotransferase; GGT, gamma glutamyl transferase; ALP, alkaline phosphatase; Tbil, total bilirubin; ALB, albumin; BMI, body mass index; WC, waist circumference; HC, hip circumference; WHtR, waist-to-height ratio; WHR, waist-to-hip ratio; LAP, lipid accumulation product; CMI, cardiometabolic index; BRI, body roundness index; TyG index, triglyceride-glucose (TyG) index; CI, conicity index; BAI, body adiposity index; AVI, abdominal volume index; HSI, hepatic steatosis index; VAI, visceral adiposity index.

*P-value<0.05; **P-value<0.01; ***P-value<0.001.

**Table 3:** Multivariate logistic regression models evaluating the associations of anthropometric indices with MAFLD.

	Model 1		Model 2		Model 3	
	OR (95% CI)	P-value	OR (95% CI)	P-value	OR (95% CI)	P-value
***BMI (kg/m²)***						
<25.0	1.00 (Reference)		1.00 (Reference)		1.00 (Reference)	
25.0–30.0	5.94 (4.76, 7.40)	<0.001***	5.02 (3.95, 6.37)	<0.001***	3.91 (3.01, 5.08)	<0.001***
≥30.0	18.25 (14.70, 22.65)	<0.001***	19.81 (15.53, 25.28)	<0.001***	13.65 (10.39, 17.93)	<0.001***
Per SD increment	3.55 (3.24, 3.90)	<0.001***	4.01 (3.59, 4.48)	<0.001***	3.44 (3.03, 3.90)	<0.001***
***WHtR***						
<0.5	1.00 (Reference)		1.00 (Reference)		1.00 (Reference)	
≥0.5	22.59 (15.75, 32.39)	<0.001***	16.85 (11.45, 24.80)	<0.001***	12.47 (7.95, 19.56)	<0.001***
Per SD increment	3.88 (3.53, 4.27)	<0.001***	4.25 (3.78, 4.73)	<0.001***	3.64 (3.20, 4.14)	<0.001***
***WHR***						
<0.90 in male or<0.85 in female	1.00 (Reference)		1.00 (Reference)		1.00 (Reference)	
≥0.90 in male or ≥0.85 in female	9.01 (7.26, 11.19)	<0.001***	7.32 (5.69, 9.41)	<0.001***	5.38 (4.04, 7.15)	<0.001***
Per SD increment	2.98 (2.74, 3.26)	<0.001***	3.23 (2.89, 3.61)	<0.001***	2.77 (2.44, 3.14)	<0.001***
***LAP***						
<26.157	1.00 (Reference)		1.00 (Reference)		1.00 (Reference)	
26.157–49.783	6.37 (4.86,8.34)	<0.001***	5.47 (4.07,7.35)	<0.001***	4.41 (3.17, 6.15)	<0.001***
49.783–82.597	16.66 (12.76, 21.77)	<0.001***	14.19 (10.59, 19.01)	<0.001***	10.37 (7.47, 14.39)	<0.001***
≥82.597	42.96 (32.56, 56.67)	<0.001***	35.86 (26.45, 48.60)	<0.001***	23.63 (16.74, 33.37)	<0.001***
Per SD increment	5.06 (4.48,5.72)	<0.001***	4.54 (3.97, 5.19)	<0.001***	3.79 (3.25, 4.40)	<0.001***
***CMI***						
<0.335	1.00 (Reference)		1.00 (Reference)		1.00 (Reference)	
0.335–0.589	3.83 (3.06, 4.80)	<0.001***	3.43 (2.69, 4.38)	<0.001***	2.74 (2.09, 3.60)	<0.001***
0.589–1.018	8.29 (6.64, 10.34)	<0.001***	6.91 (5.42, 8.81)	<0.001***	5.16 (3.94, 6.77)	<0.000***
≥1.018	18.00 (14.32, 22.62)	<0.001***	15.33 (11.88, 19.78)	<0.001***	9.85 (7.39, 13.13)	<0.001***
Per SD increment	3.71 (3.28, 4.20)	<0.001***	3.48 (3.03, 4.00)	<0.001***	2.83 (2.43, 3.30)	<0.001***
***TyG index***						
<8.204	1.00 (Reference)		1.00 (Reference)		1.00 (Reference)	
8.204–8.605	2.51 (2.04, 3.09)	<0.001***	2.37 (1.88, 2.99)	<0.01**	2.05 (1.57, 2.68)	<0.001***
8.605–9.055	5.41 (4.42, 6.62)	<0.001***	4.72 (3.76, 5.93)	<0.001***	3.70 (2.84, 4.81)	<0.001***
≥9.055	10.68 (8.68, 13.15)	<0.001***	9.15 (7.21, 11.62)	<0.001***	6.34 (4.82, 8.33)	<0.001***
Per SD increment	2.50 (2.28, 2.74)	<0.001***	2.50 (2.28, 2.74)	<0.001***	2.22 (2.00, 2.47)	<0.001***
***BRI***						
<3.871	1.00 (Reference)		1.00 (Reference)		1.00 (Reference)	
3.871–5.240	6.03 (4.64, 7.84)	<0.001***	4.95 (3.71, 6.60)	<0.001***	4.18 (3.03, 5.78)	<0.001***
5.240–6.969	15.73 (12.15, 20.38)	<0.001***	13.72 (10.26, 18.34)	<0.001***	10.65 (7.68, 14.78)	<0.001***
≥6.969	33.56 (25.72, 43.79)	<0.001***	37.86 (27.91, 51.35)	<0.001***	26.32 (18.61, 37.22)	<0.001***
Per SD increment	3.69 (3.35, 4.05)	<0.001***	3.92 (3.51, 4.38)	<0.001***	2.22 (2.00, 2.47)	<0.001***
***CI***						
<0.124	1.00 (Reference)		1.00 (Reference)		1.00 (Reference)	
0.124–0.131	4.38 (3.48, 5.50)	<0.001***	3.73 (2.89, 4.81)	<0.001***	3.55 (2.62, 4.80)	<0.001***
0.131–0.137	9.58 (7.63, 12.02)	<0.001***	7.99 (6.14, 10.40)	<0.001***	6.80 (4.98, 9.28)	<0.001***
≥0.137	15.15 (12.07, 19.01)	<0.001***	13.86 (10.59, 18.15)	<0.001***	10.46 (7.62, 14.36)	<0.001***
Per SD increment	3.06 (2.81, 3.34)	<0.001***	3.03 (2.73, 3.37)	<0.001***	2.58 (2.29, 2.91)	<0.001***
***BAI***						
<26.273	1.00 (Reference)		1.00 (Reference)		1.00 (Reference)	
26.273–30.222	2.31 (1.91, 2.80)	<0.001***	3.32 (2.65, 4.15)	<0.001***	2.79 (2.16, 3.61)	<0.001***
30.222–35.997	3.41 (2.82, 4.13)	<0.001***	7.81 (6.07, 10.03)	<0.001***	5.84 (3.39, 7.78)	<0.001***
≥35.997	5.51 (4.55, 6.67)	<0.001***	21.87 (16.44, 29.08)	<0.001***	13.91 (10.04, 19.28)	<0.001***
Per SD increment	1.92 (1.79, 2.06)	<0.001***	3.22 (2.89, 3.59)	<0.001***	2.65 (2.35, 3.00)	<0.001***
***AVI***						
<15.287	1.00 (Reference)		1.00 (Reference)		1.00 (Reference)	
15.287–19.405	6.42 (4.92, 8.38)	<0.001***	5.31 (3.98, 7.08)	<0.001***	4.54 (3.29, 6.25)	<0.001***
19.405–24.421	15.06 (11.58, 19.59)	<0.001***	12.95 (9.67, 17.34)	<0.001***	10.15 (7.31, 14.09)	<0.001***
≥24.421	41.13 (31.30, 54.06)	<0.001***	39.87 (29.40, 54.12)	<0.001***	26.98 (19.10, 38.10)	<0.001***
Per SD increment	4.18 (3.79, 4.62)	<0.001***	4.13 (3.69, 4.63)	<0.001***	3.54 (3.12, 4.03)	<0.001***
***HSI***						
<32.659	1.00 (Reference)		1.00 (Reference)		1.00 (Reference)	
32.659–37.483	4.74 (3.66, 6.14)	<0.001***	3.76 (2.85, 4.98)	<0.001***	3.42 (2.50, 4.67)	<0.001***
37.483–44.0	13.62 (10.62, 17.47)	<0.001***	13.02 (9.90, 17.10)	<0.001***	10.64 (7.81, 14.48)	<0.001***
≥44.0	34.52 (26.58, 44.80)	<0.001***	40.81 (30.39, 54.78)	<0.001***	30.12 (21.43, 42.34)	<0.001***
Per SD increment	4.17 (3.78, 4.59)	<0.001***	4.75 (4.23, 5.34)	<0.001***	4.12 (3.61, 4.71)	<0.001***
***VAI***					1.00 (Reference)	
<0.986	1.00 (Reference)		1.00 (Reference)			
0.986–1.621	2.46 (2.00, 3.01)	<0.001***	2.44 (1.95, 3.05)	<0.001***	2.07 (1.60, 2.67)	<0.001***
1.621–2.717	5.13 (4.20, 6.27)	<0.001***	4.88 (3.90, 6.10)	<0.001***	3.64 (2.82, 4.68)	<0.001***
≥2.717	9.24 (7.53, 11.33)	<0.001***	8.93 (7.08, 11.26)	<0.001***	6.12 (4.70, 7.97)	<0.001***
Per SD increment	2.69 (2.42, 3.00)	<0.001***	2.55 (2.26, 2.86)	<0.001***	2.15 (1.89, 2.45)	<0.001***

BMI, body mass index; WHtR, waist-to-height ratio; WHR, waist-to-hip ratio; LAP, lipid accumulation product; CMI, cardiometabolic index; BRI, body roundness index; TyG index, triglyceride-glucose (TyG) index; CI, conicity index; BAI, body adiposity index; AVI, abdominal volume index; HSI, hepatic steatosis index; VAI, visceral adiposity index.

Model 1: no covariates were adjusted.

Model 2: adjusted by gender, age, race/ethnicity, education level, marital status.

Model 3: adjusted by model 1 plus uric acid, lipid lowering medication use and antihypertensive medication use.

**P-value<0.01; ***P-value<0.001.

Subgroup analysis was performed to evaluate the robustness of the association between anthropometric and MAFLD. The analyses of the interactions between gender, age, BMI, abdominal obesity, and different anthropometric indices on MAFLD are shown in **Table 4**. Generally, the LAP (as a continuous variable) was significantly associated with MALFD risk across various subgroups. There was a significant interaction in the BMI subgroup (P for interaction <0.001 in model 3). The ORs of LAP on MALFD were more prominent in those without overweight (OR = 8.76, 95% CI: 5.07 to 15.14) than in those with overweight (OR = 2.58, 95% CI: 2.21 to 3.01). Furthermore, the interactions between AVI and gender (P for interaction = 0.0063), HSI and gender (P for interaction<0.0001), BRI and gender (P for interaction<0.0001), and WHtR and gender (P for interaction<0.0001) on MAFLD were statistically significant. Besides, the interactions between AVI and BMI (P for interaction = 0.0004), BRI and BMI (P for interaction<0.0001), and WHtR and BMI (P for interaction<0.0001), HSI and BMI (P for interaction = 0.0091) on MAFLD were statistically significant.

**Table 4:** Multivariate logistic regression models evaluating the associations of anthropometric indices with MAFLD stratified by gender, age, BMI, and abdominal obesity.

	N (%)	OR (95% CI)	P for interaction
***LAP***			
Total	4,195 (100%)	3.79 (3.25, 4.40)	
Gender			**0.7097**
Man	2,069 (49.32%)	3.89 (3.14, 4.83)	
Female	2,126 (50.68%)	3.68 (3.00, 4.52)	
Age			0.016*
<50	2,158 (51.44%)	5.10 (3.97, 6.56)	
≥50	2,037 (48.56%)	3.14 (2.60, 3.78)	
BMI			<0.0001***
<25	1,161 (27.68%)	8.76 (5.07, 15.14)	
≥25	3,034 (72.32%)	2.58 (2.21, 3.01)	
Abdominal obesity			0.0342*
No	1,799 (42.88%)	3.54 (2.59, 4.85)	
Yes	2,396 (57.12%)	2.43 (2.05, 2.89)	
***AVI***			
Total	4,195 (100%)	3.54 (3.12, 4.03)	
Gender			0.0063**
Man	2,069 (49.32%)	4.36 (3.55, 5.34)	
Female	2,126 (50.68%)	3.10 (2.65, 3.62)	
Age			**0.2455**
<50	2,158 (51.44%)	3.86 (3.20, 4.65)	
≥50	2,037 (48.56%)	3.35 (2.83, 3.95)	
BMI			0.0004***
<25	1,161 (27.68%)	9.69 (4.68, 20.04)	
≥25	3,034 (72.32%)	2.65 (2.30, 3.06)	
Abdominal obesity			<0.0001***
No	1,799 (42.88%)	10.06 (6.20, 16.31)	
Yes	2,396 (57.12%)	2.38 (2.03, 2.79)	
***HSI***			
Total	4,195 (100%)	4.12 (3.61, 4.71)	
Gender			<0.0001***
Man	2,069 (49.32%)	5.37 (4.33, 6.67)	
Female	2,126 (50.68%)	3.50 (2.98, 4.11)	
Age			**0.5843**
<50	2,158 (51.44%)	4.29 (3.53, 5.20)	
≥50	2,037 (48.56%)	4.00 (3.36, 4.76)	
BMI			0.0091**
<25	1,161 (27.68%)	7.84 (4.08, 15.08)	
≥25	3,034 (72.32%)	3.29 (2.82, 3.84)	
Abdominal obesity			<0.0001***
No	1,799 (42.88%)	6.35 (4.45, 9.07)	
Yes	2,396 (57.12%)	2.85 (2.42, 3.34)	
***WHtR***			
Total	4,195 (100%)	3.64 (3.20, 4.14)	
Gender			<0.0001***
Man	2,069 (49.32%)	5.06 (4.07, 6.29)	
Female	2,126 (50.68%)	3.03 (2.61, 3.52)	
Age			**0.1951**
<50	2,158 (51.44%)	3.99 (3.32, 4.80)	
≥50	2,037 (48.56%)	3.42 (2.89, 4.03)	
BMI			0.0043**
<25	1,161 (27.68%)	6.42 (3.60, 11.44)	
≥25	3,034 (72.32%)	2.78 (2.39, 3.22)	
Abdominal obesity			<0.0001***
No	1,799 (42.88%)	8.26 (5.28, 12.93)	
Yes	2,396 (57.12%)	2.34 (1.98, 2.75)	
***BRI***			
Total	4,195 (100%)	2.22 (2.00, 2.47)	
Gender			<0.0001***
Man	2,069 (49.32%)	5.03 (4.02, 6.30)	
Female	2,126 (50.68%)	2.74 (2.38, 3.16)	
Age			**0.2441**
<50	2,158 (51.44%)	3.64 (3.03, 4.35)	
≥50	2,037 (48.56%)	3.17 (2.70, 3.72)	
BMI			<0.0001***
<25	1,161 (27.68%)	8.32 (4.27, 16.20)	
≥25	3,034 (72.32%)	2.49 (2.17, 2.86)	
Abdominal obesity			<0.0001***
No	1,799 (42.88%)	11.27 (6.75, 18.80)	
Yes	2,396 (57.12%)	2.10 (1.81, 2.43)	

gender, age, race/ethnicity, education level, marital status, uric acid, lipid lowering medication use and antihypertensive medication use.

In the subgroup analysis stratified by gender and age, the model is not adjusted for the stratification variable itself.

LAP, lipid accumulation product; AVI, abdominal volume index; HSI, hepatic steatosis index; WHtR, waist-to-height ratio; BRI, body roundness index.

*P-value<0.05; **P-value<0.01; ***P-value<0.001.

### Diagnostic efficacy of various anthropometric indices for MAFLD

**Figure 2:**
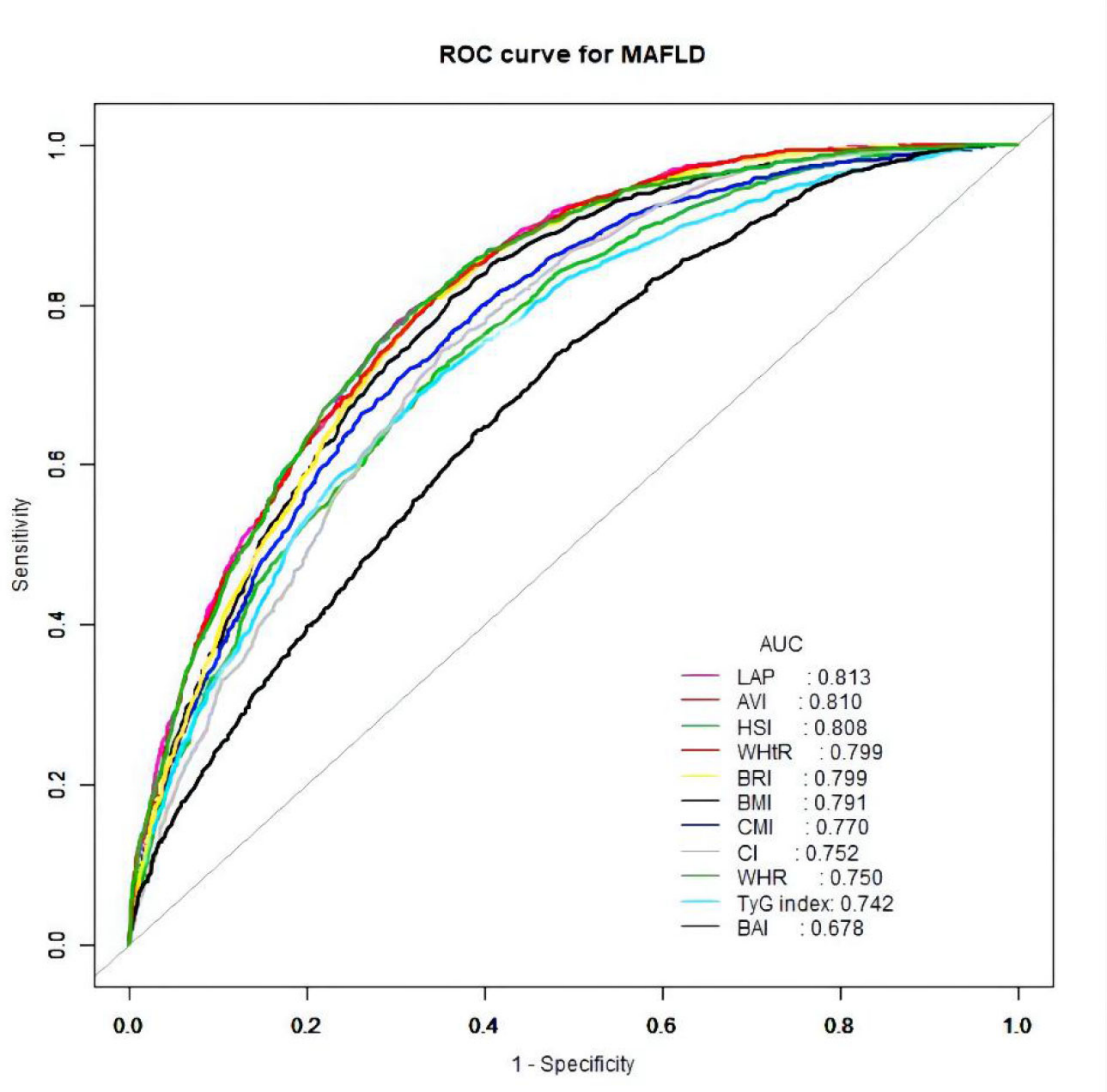
Receiver operator characteristic for anthropometric indices in diagnosing MAFLD.

**Figure 3:**
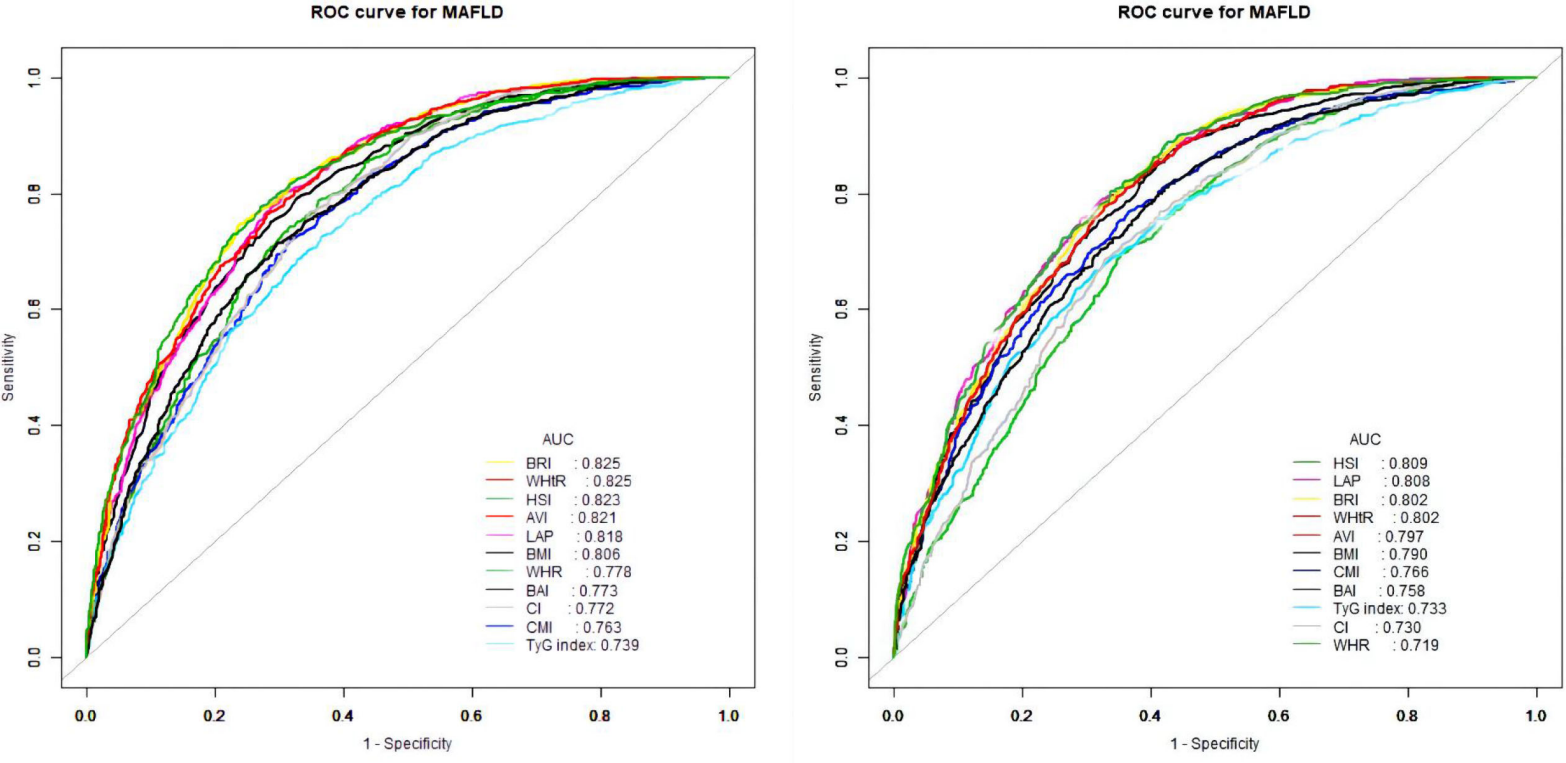
Receiver operator characteristic for anthropometric indices in diagnosing MAFLD stratified by gender [(left) males and (right) females].

**Figure 4:**
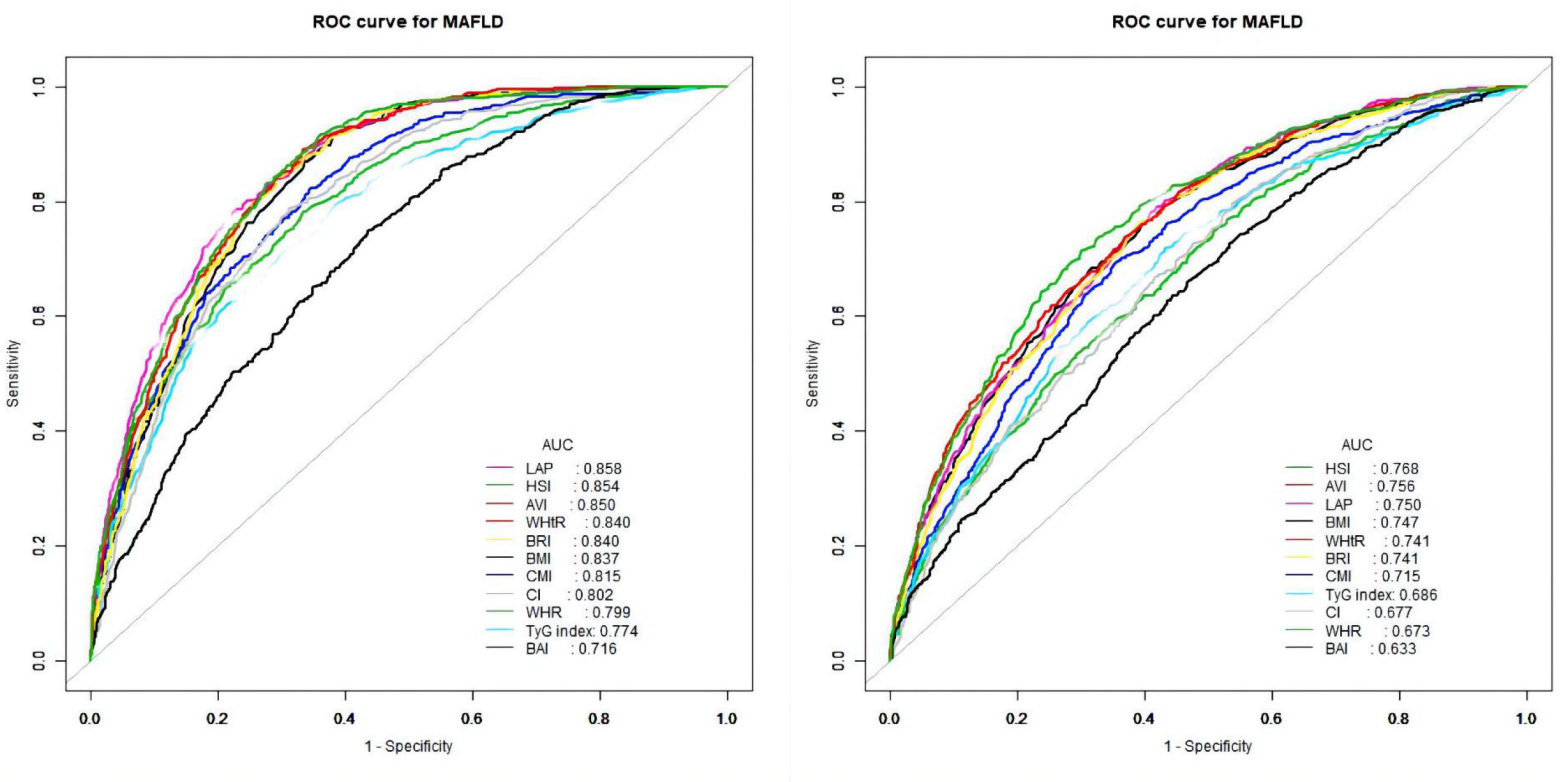
Receiver operator characteristic for anthropometric indices in diagnosing MAFLD stratified by age [(left) 18–50 years and (right) >50 years].

The ROC curves of the various anthropometric indices for diagnosing MAFLD were presented in **Figure 2**. The AUC was greatest for LAP (0.813, 95% CI: 0.800 to 0.823), followed by AVI (0.810, 95%CI: 0.797 to 0.822), HSI (0.808, 95%CI: 0.796 to 0.821), BRI (0.799, 95%CI: 0.786 to 0.812), and WHtR (0.609, 95%CI: 0.560 to 0.656). We observed good diagnostic values for LAP, BRI, and HSI for MAFLD in subgroups of age and gender (**Figures 3** and **4**). In the subgroups, BRI showed the best diagnostic value for MAFLD in men (AUC 0.825, 95% CI 0.812 to 0.886). LAP also had more favorable diagnostic performance in men than the other subgroups (AUC 0.818, 95% CI 0.803 to 0.844). As for HSI, the highest diagnostic accuracy was found in women (AUC 0.809, 95% CI 0.709 to 0.819) and in the subgroup of age >50 years (AUC 0.768, 95% CI 0.743 to 0.783). In addition, LAP showed the best AUC for diagnosing MAFLD in the subgroup (18 to 50 years) (AUC 0.858, 95% CI 0.850 to 0.868). ROC curve analysis was used to detect sex-specific LAP cut-off values for diagnosing MAFLD (**Table 5** and **Figure 3**). With the Youden method, the optimal cutoff point for diagnosing MAFLD with LAP was 47.67. At or above the 47.67 cut-off, LAP demonstrated a sensitivity of 79.2%, a specificity of 68.5%, a positive predictive value (PPV) of 65.2%, and a negative predictive value (NPV) of 81.5% for MAFLD. Besides, LAP values of ≥46.43 (AUC: 0.818) for men and ≥47.84 (AUC: 0.808) for women were the cut-off values best able to diagnose MAFLD.

**Table 5:** Diagnostic performance of anthropometric indices for the detection of MAFLD.

	AUC	AUC 95% CI low	AUC 95% CI up	Cut-off	Sp (%)	Se (%)	YI	PPV (%)	NPV (%)	PLR	NLR
**Total**											
BMI	0.791	0.778	0.805	27.45	63.3	81.3	0.446	62.3	81.9	2.215	0.295
WHtR	0.799	0.786	0.812	0.58	66.7	79.9	0.466	64.2	81.6	2.398	0.302
WHR	0.750	0.736	0.765	0.94	65.3	71.9	0.372	60.7	75.7	2.071	0.431
CMI	0.770	0.756	0.784	0.61	69.0	71.4	0.405	63.3	76.4	2.307	0.414
TyG index	0.742	0.727	0.756	8.64	67.6	68.7	0.363	61.3	74.3	2.120	0.464
LAP	0.813	0.800	0.826	47.67	68.5	79.2	0.477	65.2	81.5	2.513	0.304
BRI	0.799	0.786	0.812	5.07	66.7	79.9	0.466	64.2	81.6	2.398	0.302
CI	0.752	0.737	0.766	0.13	64.7	74.3	0.39	61.1	77.2	2.106	0.397
BAI	0.678	0.662	0.694	28.68	52.0	73.7	0.257	53.4	72.6	1.536	0.505
AVI	0.810	0.797	0.822	18.70	65.4	81.4	0.468	63.7	82.5	2.354	0.284
HSI	0.808	0.796	0.821	37.85	70.3	77.1	0.474	65.9	80.4	2.593	0.326
***Man***											
BMI	0.806	0.787	0.824	28.05	71.4	75.2	0.466	71.0	75.6	2.627	0.347
WHtR	0.825	0.808	0.843	0.59	76.4	74.5	0.509	74.6	76.3	3.158	0.334
WHR	0.779	0.759	0.798	0.97	68.7	74.1	0.428	68.7	74.0	2.365	0.377
CMI	0.764	0.743	0.784	0.68	67.9	72.3	0.402	67.7	72.5	2.254	0.408
TyG index	0.739	0.717	0.760	8.66	63.2	72.8	0.36	64.8	71.4	1.976	0.430
LAP	0.818	0.800	0.835	46.43	68.8	80.3	0.492	70.6	79.0	2.579	0.286
BRI	0.825	0.808	0.843	5.12	76.4	74.5	0.509	74.6	76.3	3.158	0.334
CI	0.772	0.752	0.792	0.13	65.2	76.8	0.42	67.3	75.2	2.208	0.355
BAI	0.773	0.753	0.793	27.14	70.4	71.3	0.417	69.2	72.5	2.412	0.407
AVI	0.821	0.804	0.839	19.85	71.7	76.5	0.483	71.6	76.7	2.708	0.327
HSI	0.823	0.806	0.841	37.24	72.7	77.8	0.505	72.6	77.9	2.848	0.305
***Female***											
BMI	0.790	0.771	0.809	26.75	56.8	87.4	0.442	54.8	88.3	2.023	0.221
WHtR	0.802	0.784	0.821	0.60	64.4	81.2	0.456	57.7	85.1	2.282	0.292
WHR	0.719	0.698	0.741	0.91	64.7	69.6	0.343	54.1	78.0	1.97	0.47
CMI	0.766	0.745	0.786	0.48	63.5	76.9	0.404	55.8	82.1	2.108	0.364
TyG index	0.733	0.712	0.755	8.59	68.7	66.6	0.353	56.0	77.5	2.129	0.486
LAP	0.808	0.79	0.826	47.84	67.7	79.0	0.467	59.4	84.4	2.444	0.310
BRI	0.802	0.784	0.821	5.36	64.4	81.2	0.456	57.7	85.1	2.282	0.292
CI	0.73	0.709	0.752	0.13	67.1	68.7	0.358	55.5	78.2	2.087	0.466
BAI	0.758	0.738	0.779	33.21	56.5	82.2	0.387	53.1	84.1	1.891	0.316
AVI	0.797	0.778	0.815	18.71	67.2	77.6	0.449	58.6	83.4	2.368	0.333
HSI	0.809	0.791	0.827	37.85	66.2	80.4	0.466	58.7	84.9	2.376	0.296
***> 50 years***											
BMI	0.748	0.727	0.768	28.65	68.3	68.5	0.368	68.7	68.1	2.161	0.462
WHtR	0.741	0.720	0.763	0.60	60.8	76.3	0.371	66.4	71.7	1.947	0.389
WHR	0.673	0.650	0.697	0.98	70.6	53.6	0.241	64.9	59.9	1.821	0.658
CMI	0.715	0.692	0.737	0.62	64.5	69.5	0.34	66.5	67.5	1.956	0.473
TyG index	0.686	0.663	0.709	8.64	56.9	71.8	0.287	62.9	66.5	1.666	0.496
LAP	0.750	0.730	0.771	47.67	58.2	79.0	0.372	65.8	73.1	1.89	0.361
BRI	0.741	0.720	0.763	5.33	60.8	76.3	0.371	66.4	71.7	1.947	0.389
CI	0.677	0.654	0.7	0.13	46.2	79.7	0.259	60.1	69.1	1.482	0.440
BAI	0.633	0.609	0.657	28.41	45.4	74.1	0.195	58.0	63.3	1.356	0.571
AVI	0.756	0.736	0.777	19.77	63.2	73.8	0.37	67.1	70.3	2.004	0.415
HSI	0.768	0.747	0.788	37.83	69.9	71.4	0.413	70.7	70.6	2.371	0.41
***18-50 years***											
BMI	0.837	0.821	0.854	26.85	62.0	91.4	0.534	57.0	92.9	2.405	0.139
WHtR	0.841	0.824	0.857	0.57	70.6	84.2	0.548	61.2	89.0	2.866	0.224
WHR	0.799	0.780	0.818	0.91	66.7	78.1	0.447	56.3	84.7	2.342	0.329
CMI	0.816	0.798	0.834	0.51	65.7	82.1	0.478	56.8	87.0	2.391	0.272
TyG index	0.774	0.754	0.795	8.42	62.0	79.0	0.41	53.4	84.3	2.078	0.339
LAP	0.858	0.842	0.873	49.24	77.3	78.6	0.559	65.6	86.8	3.462	0.277
BRI	0.841	0.824	0.857	4.75	70.6	84.2	0.548	61.2	89.0	2.866	0.224
CI	0.802	0.783	0.821	0.13	68.8	78.7	0.475	58.2	85.5	2.525	0.309
BAI	0.716	0.694	0.737	28.78	56.3	74.9	0.313	48.6	80.3	1.716	0.445
AVI	0.851	0.835	0.866	17.43	66.6	89.0	0.556	59.5	91.7	2.665	0.165
HSI	0.854	0.838	0.870	36.03	63.9	91.6	0.556	58.3	93.3	2.541	0.131

Cut-off values obtained required maximal Youden index.

AUC, area under the receiver operational characteristics curve; CI, confidence interval; Sp, Specificity; Se, Sensitivity; YI, Youden index; PPV, positive predictive value; NPV, negative predictive value; PLR, positive likelihood ratio; NLR, negative likelihood ratio.

### Internal validation

We randomly divided all participants into the development group (n = 2,097) and the validation group (n = 2,098). **Table 6** illustrates the characteristics of the participants of the training and validation groups. Besides, **Table 7** shows the characteristics of the two groups divided by MAFLD status. Participants with MAFLD had higher age, BMI, waist circumference, hip circumference, TG, TC, HDL-c, AST, ALT, GGT, ALP, SBP, DBP, BNN, uric acid, anthropometric indices, and higher rates of males, diabetes, prediabetes, insulin resistance, lipid-lowering drug use, and blood pressure lowering drug use in the development and validation groups. In contrast, participants in the MAFLD group had lower levels of HDL-c. Furthermore, in **Table 8**, we found that in the development group, LAP showed the greatest AUC (0.812; 95% CI: 0.800–0.835). Besides, the AUC of LAP remained the highest in the validation group (0.809; 95% CI: 0.791–0.827).

**Table 6:** Baseline characteristics of the development and validation group.

Characteristic	Development group	Validation group	P-value
N	2,097	2,098	
Age	45.4 ± 0.73	45.4 ± 0.68	0.992
Sex			0.224
Male	48.06 ± 1.58	48.84 ± 1.66	
Female	51.94 ± 1.58	51.16 ± 1.66	
Race/ethnicity			0.013*
Mexican American	9.87 ± 1.80	9.69 ± 1.87	
Other Hispanic	6.13 ± 0.71	8.13 ± 1.11	
Non-Hispanic White	60.90 ± 2.58	61.02 ± 2.81	
Non-Hispanic Black	10.79 ± 1.61	11.26 ± 1.71	
Mixed non‐Hispanic	12.31 ± 1.50	9.90 ± 1.34	
Education level (%)			0.664
High school and below	39.24 ± 1.89	38.47 ± 2.25	
Higher than high school	60.76 ± 1.89	61.53 ± 2.25	
Marital status			0.602
Married or living with partner	62.05 ± 1.48	62.90 ± 1.87	
Widowed/Divorced/Separated/Never married	37.95 ± 1.48	37.10 ± 1.87	
Lipid lowering medication use	21.88 ± 1.46	22.43 ± 1.54	0.705
Antihypertensive medication use	25.92 ± 2.05	25.14 ± 1.45	0.703
Drinking status (%)			0.840
Never drinkers	7.79 ± 1.11	7.35 ± 0.97	
Current drinkers	77.41 ± 1.29	76.65 ± 1.53	
Former drinkers	14.80 ± 1.41	16.00 ± 1.42	
Smoking status (%)			0.752
Never smokers	58.73 ± 2.60	59.60 ± 1.92	
Current smokers	17.92 ± 1.74	16.32 ± 1.48	
Former smokers	23.35 ± 1.62	24.08 ± 1.37	
Height (cm)	168.11 ± 0.35	168.36 ± 0.43	0.675
Weight (kg)	83.71 ± 1.10	84.33 ± 0.96	0.612
Body mass index	29.54 ± 0.36	29.62 ± 0.34	0.835
Waist circumference (cm)	99.86 ± 0.87	100.32 ± 0.90	0.581
Hip circumference (cm)	107.32 ± 0.68	107.36 ± 0.54	0.961
Hypertension (%)	40.54 ± 1.96	39.35 ± 1.93	0.570
Diabetes (%)	13.00 ± 0.75	13.90 ± 0.75	0.355
Prediabetes (%)	34.77 ± 1.59	34.26 ± 1.73	0.837
Overweight (%)	70.84 ± 1.90	73.41 ± 1.68	0.234
Abdominal obesity (%)	57.03 ± 2.14	58.48 ± 2.26	0.564
Insulin resistance (%)	21.20 ± 0.87	20.67 ± 1.26	0.688
High sensitivity CRP (%)	45.68 ± 1.85	47.11 ± 2.03	0.457
MAFLD (%)	40.04 ± 1.84	41.75 ± 1.19	0.375
Lipid profile			
Total cholesterol (mg/dl)	189.33 ± 1.96	187.49 ± 1.79	0.323
Triglyceride (mg/dl)	140.18 ± 4.22	141.01 ± 3.45	0.840
High-density cholesterol (mg/dl)	53.71 ± 0.68	52.99 ± 0.58	0.366
Low-density cholesterol (mg/dl)	110.94 ± 1.91	110.06 ± 2.03	0.713
Systolic blood pressure (mmHg)	120.85 ± 0.51	121.38 ± 0.50	0.420
Diastolic blood pressure (mmHg)	74.19 ± 0.45	74.15 ± 0.48	0.941
ALT (IU/L)	23.57 ± 0.63	23.49 ± 0.53	0.916
AST (IU/L)	22.36 ± 0.38	22.49 ± 0.39	0.795
GGT (IU/L)	29.86 ± 1.20	29.91 ± 0.96	0.976
ALP (IU/L)	76.83 ± 0.96	76.12 ± 0.77	0.557
Total bilirubin (mg/dl)	0.47 ± 0.01	0.48 ± 0.01	0.385
Albumin (g/dl)	4.13 ± 0.02	4.09 ± 0.02	0.095
Bun (mg/dl)	14.45 ± 0.22	14.56 ± 0.16	0.546
Serum creatinine (mg/dl)	0.86 ± 0.01	0.86 ± 0.01	0.253
Uric acid (mg/dl)	5.36 ± 0.04	5.38 ± 0.05	0.633
Obesity-related indices			
WHtR	0.60 ± 0	0.60 ± 0.01	0.784
WHR	0.93 ± 0	0.93 ± 0	0.344
LAP	65.24 ± 3.00	67.34 ± 3.15	0.444
CMI	0.83 ± 0.04	0.87 ± 0.04	0.325
BRI	5.54 ± 0.12	5.55 ± 0.13	0.921
TyG index	8.65 ± 0.03	8.65 ± 0.02	0.839
CI	0.13 ± 0	0.13 ± 0	0.480
BAI	31.55 ± 0.34	31.43 ± 0.35	0.766
AVI	20.65 ± 0.37	20.83 ± 0.38	0.623
HSI	39.04 ± 0.43	39.06 ± 0.45	0.958
VAI	2.20 ± 0.08	2.25 ± 0.08	0.551

Continuous data are shown as the mean ± SD and categorical data as n (%).

ALT, alanine aminotransferase; AST, aspartate aminotransferase; GGT, gamma glutamyl transferase; ALP, alkaline phosphatase; BUN, blood urea nitrogen; WHtR, waist-to-height ratio; WHR, waist-to-hip ratio; LAP, lipid accumulation product; CMI, cardiometabolic index; BRI, body roundness index; TyG index, triglyceride-glucose (TyG) index; CI, conicity index; BAI, body adiposity index; AVI, abdominal volume index; HSI, hepatic steatosis index; VAI, visceral adiposity index.

*P-value<0.05.

**Table 7:** Baseline characteristics for the training and validation groups by MAFLD status.

Characteristic	Development group		Validation group	
	MAFLD (+)	MAFLD (−)	MAFLD (+)	MAFLD (−)
N	887	1,220	916	1,182
Age	48.6 ± 0.9	43.27 ± 0.75	49.48 ± 0.85	42.47 ± 0.89
Sex				
Male	52.86 ± 2.07	44.86 ± 1.88	59.36 ± 2.15	45.28 ± 2.28
Female	47.14 ± 2.07	55.14 ± 1.88	40.64 ± 2.15	54.72 ± 2.28
Race/ethnicity				
Mexican American	14.29 ± 2.52	6.91 ± 1.43	13.15 ± 3.06	7.21 ± 1.27
Other Hispanic	6.29 ± 1.1	6.03 ± 0.98	6.77 ± 0.98	9.11 ± 1.66
Non-Hispanic White	58.33 ± 3.02	62.63 ± 2.94	60.73 ± 3.6	61.22 ± 3.05
Non-Hispanic Black	9.59 ± 1.62	11.59 ± 1.75	8.94 ± 1.79	12.93 ± 1.71
Mixed non‐Hispanic	11.5 ± 1.79	12.85 ± 1.84	10.41 ± 1.86	9.54 ± 1.35
Education level (%)				
High school and below	40.41 ± 1.84	38.43 ± 3.23	42.21 ± 3.27	35.7 ± 2.35
Higher than high school	59.59 ± 1.84	61.57 ± 3.23	57.79 ± 3.27	64.3 ± 2.35
Marital status				
Married or Living with partner	68.23 ± 1.89	57.74 ± 1.75	69.75 ± 2.92	57.81 ± 2.37
Widowed/Divorced/Separated/Never married	31.77 ± 1.89	42.26 ± 1.75	30.25 ± 2.92	42.19 ± 2.37
Lipid lowering medication use	28.68 ± 2.82	17.34 ± 1.82	34.71 ± 2.6	13.62 ± 1.53
Antihypertensive medication use	37.81 ± 3.01	17.98 ± 2.15	14.96 ± 1.53	25.92 ± 2.05
Drinking status (%)				
Never drinkers	8.37 ± 2.26	7.4 ± 1.16	7.66 ± 1.27	7.13 ± 1.19
Current drinkers	76.21 ± 2.32	78.22 ± 1.37	75.35 ± 1.77	77.57 ± 1.99
Former drinkers	15.43 ± 1.93	14.38 ± 1.54	16.99 ± 1.56	15.31 ± 1.78
Smoking status (%)				
Never smokers	57.77 ± 2.84	59.37 ± 2.96	55.99 ± 2.67	62.2 ± 2.6
Current smokers	17.11 ± 2.07	18.46 ± 2.02	14.78 ± 1.82	17.43 ± 1.93
Former smokers	25.11 ± 2.53	22.17 ± 2.13	29.24 ± 2.58	20.38 ± 1.64
Height (cm)	168.65 ± 0.35	167.75 ± 0.42	169.78 ± 0.62	167.34 ± 0.46
Weight (kg)	97.30 ± 1.19	74.62 ± 1.05	97.32 ± 1.62	75.02 ± 0.69
Body mass index	34.13 ± 0.44	26.48 ± 0.35	33.69 ± 0.53	26.7 ± 0.27
Waist circumference (cm)	111.91 ± 0.93	91.81 ± 0.83	111.55 ± 1.34	92.27 ± 0.75
Hip circumference (cm)	115.08 ± 0.97	102.14 ± 0.69	114.37 ± 0.86	102.33 ± 0.39
Diabetes (%)	22.89 ± 1.73	6.39 ± 0.69	25.09 ± 1.51	5.88 ± 0.72
Prediabetes (%)	47.07 ± 2.52	26.56 ± 1.61	48.24 ± 2.23	24.24 ± 2.09
Overweight (%)	95.4 ± 1.31	54.43 ± 2.59	95.59 ± 1.05	57.52 ± 2.46
Abdominal obesity (%)	85.26 ± 1.79	38.17 ± 2.91	83.81 ± 2.54	40.33 ± 2.68
Insulin resistance (%)	35.05 ± 1.98	11.96 ± 1.06	32.29 ± 2.83	12.34 ± 1.28
High sensitivity CRP (%)	62.52 ± 2.39	34.41 ± 2.31	62.72 ± 2.46	35.91 ± 2.22
Lipid profile				
Total cholesterol (mg/dl)	193.79 ± 3.39	186.34 ± 1.68	191.44 ± 2.07	184.66 ± 1.85
Triglyceride (mg/dl)	180.81 ± 6.47	113.04 ± 2.90	180.55 ± 6.33	112.68 ± 2.72
High-density cholesterol (mg/dl)	48.59 ± 0.76	57.13 ± 0.75	47.38 ± 0.75	57.01 ± 0.71
Low-density cholesterol (mg/dl)	113.93 ± 4.00	108.9 ± 2.31	113.89 ± 3.41	107.69 ± 1.53
Systolic blood pressure (mmHg)	123.75 ± 0.58	118.91 ± 0.7	124.2 ± 0.97	119.28 ± 0.29
Diastolic blood pressure (mmHg)	77.18 ± 0.43	72.18 ± 0.56	76.87 ± 0.78	72.13 ± 0.34
ALT (IU/L)	28.49 ± 1.08	20.28 ± 0.78	28.27 ± 1.07	20.07 ± 0.49
AST (IU/L)	23.47 ± 0.78	21.62 ± 0.67	23.66 ± 0.7	21.64 ± 0.43
GGT (IU/L)	37.48 ± 2.09	24.77 ± 1.74	37.21 ± 1.71	24.68 ± 1.28
ALP (IU/L)	80.01 ± 1.28	74.7 ± 1.66	79.89 ± 1.27	73.42 ± 0.88
Total bilirubin (mg/dl)	0.45 ± 0.01	0.48 ± 0.01	0.46 ± 0.02	0.49 ± 0.02
Albumin (g/dl)	4.08 ± 0.02	4.16 ± 0.02	4.06 ± 0.02	4.11 ± 0.02
Bun (mg/dl)	14.71 ± 0.24	14.29 ± 0.25	15.33 ± 0.32	14.02 ± 0.12
Serum creatinine (mg/dl)	0.88 ± 0.02	0.85 ± 0.01	0.89 ± 0.01	0.87 ± 0.01
Uric acid (mg/dl)	5.80 ± 0.05	5.07 ± 0.05	5.82 ± 0.07	5.07 ± 0.06
Obesity-related indices				
WHtR	0.66 ± 0.01	0.55 ± 0	0.66 ± 0.01	0.55 ± 0.01
WHR	0.97 ± 0	0.9 ± 0	0.98 ± 0.01	0.9 ± 0
LAP	101.59 ± 4.14	40.96 ± 1.88	101.98 ± 5.93	42.52 ± 1.78
CMI	1.26 ± 0.06	0.55 ± 0.02	1.29 ± 0.08	0.58 ± 0.02
BRI	7.15 ± 0.16	4.47 ± 0.11	6.96 ± 0.2	4.54 ± 0.11
TyG index	8.97 ± 0.04	8.44 ± 0.02	8.99 ± 0.03	8.4 ± 0.02
CI	0.14 ± 0	0.13 ± 0	0.14 ± 0	0.13 ± 0
BAI	34.91 ± 0.51	29.3 ± 0.37	34.06 ± 0.51	29.54 ± 0.3
AVI	25.57 ± 0.44	17.37 ± 0.33	25.39 ± 0.59	17.56 ± 0.28
HSI	44.94 ± 0.51	35.1 ± 0.42	44.39 ± 0.72	35.24 ± 0.32
VAI	3.11 ± 0.13	1.6 ± 0.05	3.12 ± 0.15	1.63 ± 0.05

Continuous data are shown as the mean ± SD and categorical data as n (%).

ALT, alanine aminotransferase; AST, aspartate aminotransferase; GGT, gamma glutamyl transferase; ALP, alkaline phosphatase; BUN, blood urea nitrogen; WHtR, waist-to-height ratio; WHR, waist-to-hip ratio; LAP, lipid accumulation product; CMI, cardiometabolic index; BRI, body roundness index; TyG index, triglyceride-glucose (TyG) index; CI, conicity index; BAI, body adiposity index; AVI, abdominal volume index; HSI, hepatic steatosis index; VAI, visceral adiposity index.

**Table 8:** The diagnostic performance of anthropometric indices obtained from development and validation group.

Anthropometric indices	Development group (N = 2,197)			Validation group (N = 2,098)		
	AUC (95 CI%)	Sensitivity	Specificity	AUC (95 CI%)	Sensitivity	Specificity
BMI	0.796 (0.777–0.815)	0.636	0.818	0.787 (0.768–0.806)	0.601	0.842
WHtR	0.807 (0.789–0.825)	0.675	0.803	0.791 (0.773–0.810)	0.668	0.790
WHR	0.749 (0.728–0.769)	0.653	0.720	0.752 (0.732–0.773)	0.657	0.718
CMI	0.769 (0.749–0.789)	0.595	0.815	0.772 (0.752–0.792)	0.752	0.659
TyG index	0.737 (0.716–0.759)	0.632	0.726	0.746 (0.726–0.767)	0.695	0.677
LAP	0.817 (0.799–0.835)	0.700	0.787	0.809 (0.791–0.827)	0.684	0.789
BRI	0.807 (0.789–0.825)	0.675	0.803	0.791 (0.773–0.810)	0.668	0.790
CI	0.755 (0.735–0.776)	0.658	0.734	0.748 (0.727–0.768)	0.617	0.774
BAI	0.689 (0.667–0.712)	0.512	0.766	0.666 (0.643–0.689)	0.561	0.682
AVI	0.815 (0.797–0.833)	0.660	0.820	0.805 (0.786–0.823)	0.616	0.845
HSI	0.811 (0.793–0.829)	0.642	0.844	0.706 (0.788–0.824)	0.702	0.764

AUC, area under the receiver operational characteristics curve; CI.

## Discussion

With the increasing trends of obesity and T2DM, the prevalence of MAFLD in the United States has increased dramatically over the past decade, from 34.4% to 38.1% (34). Notably, the association between IR and MAFLD appears to be bidirectional. On the one hand, liver lipid accumulation can lead to IR through DAG-mediated activation of PKCϵ (35). On the other hand, leptin therapy can resolve hepatic steatosis and ultimately improve insulin function (36). In a biopsy-proven NAFLD study, a portion of lean people may also develop MAFLD, which may be related to visceral fat obesity (37). Intriguingly, IR incidence in this subset of lean NAFLD patients was similar to that of NAFLD patients with obesity. Although BMI and waist circumference are widely used to assess obesity, they are limited in their ability to distinguish fat mass, lean body mass, visceral, and subcutaneous fat deposits in the abdomen. Early diagnosis of MAFLD seems possible by identifying sensitive indicators that represent insulin resistance or central obesity. To our knowledge, our study is the first to assess the value of these novel metabolic indices in the diagnosis of MAFLD in the general population of the United States.

In the NHANES 2017–2018 study, we found that novel metabolic indices such as LAP, AVI, HSI, BRI, and VAI can serve as strong risk markers for diagnosing MAFLD compared with traditional anthropometric and lipid measures in both sexes. We found that all of these metabolic indices were independently associated with an increased risk of MAFLD, irrespective of whether it was treated as a continuous or categorical variable. The LAP showed the highest OR values in both univariate and multivariate logistic regression models. In addition, LAP still showed the largest AUC value in ROC analysis, meaning that LAP is the most valuable diagnostic indicator for MAFLD.

The lipid accumulation products (LAPs) were originally developed as an indicator that performed better than BMI in identifying U.S. adults at cardiovascular risk (28). More recently, studies have shown that LAP is strongly associated with metabolic syndrome, type 2 diabetes, and nonalcoholic fatty liver disease (38–40). Studies in different countries and populations have demonstrated that LAP is a powerful tool for identifying non-alcoholic fatty liver disease. Koehler et al. enrolled 2,652 participants in the Rotterdam study and verified LAP in the diagnosis of NAFLD with a ROC of 0.786 (95% CI, 0.769 to 0.804) (41). Similar results were observed in the general population of the United States (AUC 0.741, 95% CI, 0.723 to 0.758) (38). In China, several studies have shown that LAP exhibited high diagnostic accuracy for identifying NAFLD, and the areas under the curves (AUC) were higher than 0.80 (42–44). Several cross-sectional and prospective studies have confirmed that patients with MAFLD have worse clinical indicators and outcomes than those with NAFLD, suggesting considerable clinical differences between the two diseases (10, 11). However, few studies have investigated the association between LAP and MAFLD, and the existing ones only focused on men (42). Cai et al. showed that LAP had the highest diagnostic value in MAFLD (AUC 0.868, 95% CI, 0.853 to 0.883), with a cut-off value of 24.49, compared with other anthropometric indicators (42). This is consistent with our findings. Nevertheless, the optimal LAP cut-off point (47.67) for the diagnosis of MAFLD was significantly higher than in the Chinese population. There are two possible reasons for this. On the one hand, the criteria for diagnosing abdominal obesity in the American population differ from those in China. In addition, LAP was calculated based on triglycerides and waist circumference, both of which were significantly higher in the population observed in this study than in the Chinese population. Besides, Lin et al. (22) found that the interaction between LAP and gender on NAFLD was statistically significant (p for interaction = 0.001). Although this result has not been verified in our study (p for interaction = 0.5931), we found the interactions between AVI, HSI, WHtR, and BRI and gender on MAFLD existed. Subsequently, the interactions of AVI, HSI, WHtR, and BRI with gender in MAFLD continuously existed in the younger subgroup (aged 18 to 50 years) but disappeared in the older subgroup (aged over 50 years). It means that the role of gender and hormones in MAFLD should be highlighted, which could probably uncover the important observation behind masks.

HSI, BRI, AVI, and WHtR are closely related to insulin resistance (IR) and central obesity (24, 45, 46). In addition, insulin sensitivity is higher in women, which may result in a lower prevalence of metabolic diseases in women (47). Besides, most of the total body fat of a woman (80%–90%) is stored in subcutaneous depots, especially in the gluteal–femoral fat depots, which can protect the impairments from glucose–insulin homeostasis and hypertriglyceridemia (48, 49). However, in men, body fat tends to be concentrated in visceral fat, which is closely associated with central obesity (48). Numerous studies have demonstrated the protective effect of estrogen on fat accumulation (50, 51). Women, especially premenopausal women, are protected from the adverse consequences of excess fat storage by a regional fat distribution that differs from men. The Chinese study confirmed that the prevalence of NAFLD was significantly higher in men than in women under 50 years of age (22.4% *versus* 7.1%, p<0.001), and the results were then reversed in the over-50-year-old population (52). A cross-sectional study with 508 biopsy-proven NASH showed that men are at a higher risk of having more severe liver fibrosis compared to premenopausal women, while post-menopausal women have an analogical severity of liver fibrosis compared to men (53). In addition, compared with postmenopausal women not being treated with hormone replacement therapy (HRT), postmenopausal women who received HRT had a lower prevalence of NAFLD (54). Collectively, the data indicate that the female-specific estrogen and regional fat distribution may result in the sex difference in MAFLD.

Recent studies have shown that AVI and BRI are favorable diagnostic indicators for NAFLD. Lin et al. recruited 1,969 participants (764 males and 1,205 females) in Taiwan to explore gender differences in the relationship between AVI, HSI, WHtR, BRI, and NAFLD (22). They found that AVI (AUC [0.700 in men, 0.724 in women]) and BRI (AUC [0.670 in men, 0.735 in women]) performed moderately in the diagnosis of MAFLD, with no gender interaction. Sheng et al. conducted a secondary analysis using NAGALA data to explore the relationship between anthropometric indicators and NAFLD (55). BRI was found to be an excellent predictor of NAFLD, with an AUC of 0.8156 in men and 0.8790 in women, respectively. Whether male or female, the optimal threshold of BRI in diagnosing NAFLD is around 2.87.

WHtR, the ratio of WC (cm) to height (cm), is an easily calculated indicator. Cai et al. showed that WHtR (AUC 0.863, 95% CI, 0.848 to 0.879) and AVI (AUC 0.859, 95% CI, 0.843 to 0.874) had an outstanding diagnostic value for MAFLD compared with BMI (AUC: 0.846, 95% CI, 0.829 to 0.864) in the western Chinese male population. Xie et al. (56) found that BMI and BRI had the same diagnostic ability (AUC = 0.849) for NAFLD in men, followed by WHtR (AUC = 0.810). BRI also had the best diagnostic ability in females (AUC = 0.849), followed by WHtR (AUC = 0.846). Nevertheless, some key confounders were not adjusted in the regression analysis, so the results of the study are worth discussing.

Initially developed by Lee et al. based on the Korean population, HSI is a combination of liver enzymes and BMI (24). Lin et al. showed that HSI had the greatest AUC in both men and women, 0.785 in men and 0.80 in women, respectively (22). In addition, Shang et al. found that HSI and LAP showed better diagnostic performance for NAFLD than BMI and WC (55). Considering the difference in AUC between these indicators in diagnosing NAFLD is limited, and it is impossible to know whether there is a significant difference between these results, this result needs to be treated with caution.

The present study has several strengths. Being based on data obtained from NHANES survey cycles, it provides representative data that can be generalized to the entire US multiethnic adult population aged 18 years or older. Besides, the acquisition of clinical, laboratory, and anthropometric data were standardized and homogenous. Furthermore, after adjusting for major confounders, we found that those anthropometric indices were independently associated with MAFLD, regardless of whether it was treated as a continuous or categorical variable. Additionally, we compared the diagnostic power of 12 anthropometric measures in individuals with MAFLD within the United States adult population and identified LAP as the best diagnostic predictor of MAFLD.

On the same note, several limitations should also be acknowledged. First, the cross-sectional design failed to establish temporal relationships or causality between those anthropometric indices and MAFLD. Although menopausal status could be identified in this study, the lack of estrogen examination results. Therefore, we could not survey the estrogen effect.

## Conclusion

Our results illustrated that those anthropometric indices in the present study were significantly associated with MAFLD. HSI, BRI, AVI, and WHtR were associated with MAFLD more clearly in men than in women. The lipid accumulation product is a powerful tool to diagnose metabolic dysfunction-associated fatty liver disease in the United States adults. At the same time, considering that LAP is simple to calculate and clinically available, it is suggested to use LAP as a reliable indicator for the diagnosis of MAFLD in the future.

## Data availability statement

Some or all data generated or analyzed during this study are included in this published article or in the data repositories listed in References. NHANES data is available publicly at NHANES - National Health and Nutrition Examination Survey Homepage (cdc.gov).

## Ethics statement

The protocols of NHANES were approved by the institutional review board of the National Center for Health Statistics, CDC (https://www.cdc.gov/nchs/nhanes/irba98.htm). NHANES has obtained written informed consent from all participants.

## Author contributions

RL, YZ, HCL and HJL made substantial contributions to the conception and design of the study. HJL analyzed the data. HJL and HCL drafted the manuscript. All authors contributed to the article and approved the submitted version.

## Funding

This work was supported by the Guangzhou Science and Technology Project (No. 202102010084, No. 202201010871), the Guangzhou Basic and Applied Research Foundation (No. 2021A1515111148), and the Medical Science and Technology Research Foundation of Guangdong Province (No. 20211117145113732).

## Conflict of interest

The authors declare that the research was conducted in the absence of any commercial or financial relationships that could be construed as a potential conflict of interest.

## Publisher’s note

All claims expressed in this article are solely those of the authors and do not necessarily represent those of their affiliated organizations, or those of the publisher, the editors and the reviewers. Any product that may be evaluated in this article, or claim that may be made by its manufacturer, is not guaranteed or endorsed by the publisher.
